# Chromophoric Dendrimer-Based Materials: An Overview of Holistic-Integrated Molecular Systems for Fluorescence Resonance Energy Transfer (FRET) Phenomenon

**DOI:** 10.3390/polym13244404

**Published:** 2021-12-15

**Authors:** Sebastián Bonardd, David Díaz Díaz, Angel Leiva, César Saldías

**Affiliations:** 1 Departamento de Química Orgánica, Universidad de La Laguna, Avda. Astrofísico Francisco Sánchez S/N, La Laguna, 38206 Tenerife, Spain; sbonardd@ull.edu.es (S.B.); ddiazdiaz@ull.es (D.D.D.); 2 Instituto Universitario de Bio-Orgánica Antonio González, Universidad de La Laguna, Avda. Astrofísico Francisco Sánchez 2, La Laguna, 38206 Tenerife, Spain; 3 Institutfür Organische Chemie, Universität Regensburg, Universitätsstr. 31, 93053 Regensburg, Germany; 4 Departamento de Química Física, Facultad de Química y de Farmacia, Pontificia Universidad Católica de Chile, Macul, Santiago, CL 7820436, USA; aleivac@uc.cl

**Keywords:** chromophoric dendrimers, FRET, holistic molecular systems

## Abstract

Dendrimers (from the Greek dendros → tree; meros → part) are macromolecules with well-defined three-dimensional and tree-like structures. Remarkably, this hyperbranched architecture is one of the most ubiquitous, prolific, and recognizable natural patterns observed in nature. The rational design and the synthesis of highly functionalized architectures have been motivated by the need to mimic synthetic and natural-light-induced energy processes. Dendrimers offer an attractive material scaffold to generate innovative, technological, and functional materials because they provide a high amount of peripherally functional groups and void nanoreservoirs. Therefore, dendrimers emerge as excellent candidates since they can play a highly relevant role as unimolecular reactors at the nanoscale, acting as versatile and sophisticated entities. In particular, they can play a key role in the properties of light-energy harvesting and non-radiative energy transfer, allowing them to function as a whole unit. Remarkably, it is possible to promote the occurrence of the FRET phenomenon to concentrate the absorbed energy in photoactive centers. Finally, we think an in-depth understanding of this mechanism allows for diverse and prolific technological applications, such as imaging, biomedical therapy, and the conversion and storage of light energy, among others.

## 1. Introduction

Undoubtedly, the design and development of diverse technological strategies oriented to provide rational alternatives to address the current energy problems in a timely manner is a high-priority issue at present [1,2,3,4,5]. Importantly, the exponential evolution of the energy demand, as well as the imminent depletion of traditional energy sources, along with contamination problems, have motivated the search for innovative solutions both in basic and applied science to exploit non-conventional and renewable energy sources (i.e., solar, wind, geothermal, ocean-wave) [6,7,8,9]. For example, the sun emits energy at a rate of 3.8 × 10^23^ kW s^−1^ [10]. A considerable fraction of this total, approximately 1.08 × 10^14^ kW s^−1^, reaches the surface of Earth. If 0.1% of this energy could be converted at an efficiency of 10%, it would be four times the world’s total annual generating capacity (~3000 GW). Remarkably, the total annual solar radiation striking the Earth is more than 7500 times the world’s total annual primary energy consumption (~450 EJ) [11,12]. In recent decades, the possibility of profiting from light energy has attracted much attention because this type of energy is abundant, clean, and highly available [13,14,15,16]. However, the fabrication of devices that allow for highly efficient exploitation of light energy is an aspect that remains largely unresolved [17,18,19]. Considering these backgrounds, the molecular design and the potentially adequate performance of photoactive environments emerge as promissory candidates that could play a key role in light-energy processing [20,21,22]. Specifically, molecular environments susceptible to photo-induced energy transfer could have a high potential of application to mimic nature’s behavior, e.g., the photosynthesis process [23,24]. Thus, the rigorous observation of the highly complex systems that make up nature has inspired numerous researchers to seek alternatives for the design of mimicking artificial systems [25,26]. In recent years, numerous scientific efforts have been carried out to fabricate artificial systems that are more efficient in photo-induced processes by a complete understanding of related processes to energy transfer [27,28].

**Figure 1:**
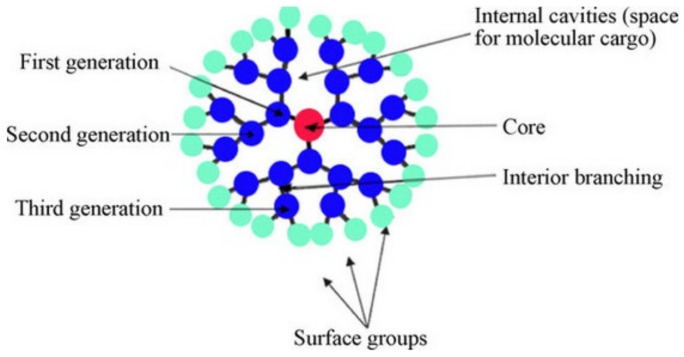
Representative illustration of the general structure of dendrimers. Reprinted with permission from [32]. Copyright 2010 Elsevier.

In this context, dendrimer structures displaying unique architectures, such as regular and hierarchical branches with many functional end groups coming from a single core, are very interesting candidates for numerous technological applications (Figure 1) [29,30,31,32].

According to these attributes, photoactive molecules or entities can be precisely placed, e.g., at the core or periphery, depending on the functionality and generation of the dendrimer. The unique structure of the dendrimers (precisely a tree-like chemical fractal architecture) allows for the mimicking of nature systems by incorporating photoactive molecules that actas photonic antennae spatially close to the reaction sites [33,34,35,36]. Remarkably, the nanoreservoirs present in the dendrimer structure (similar to living organism enzymes) provide convenient sites ideally adequate to the host, control the size, and avoid the aggregation of metal or semiconductor nanoparticles [37,38]. This singular environment can contribute to promoting and stabilizing the charge separation in different chemical species [39,40]. Considering this, it is possible to depict the relevance of the advances focused on improving the efficiency of light-induced processes for energy transfer.

## 2. Natural and Artificial Holistic-Integrated Molecular Systems

**Figure 2:**
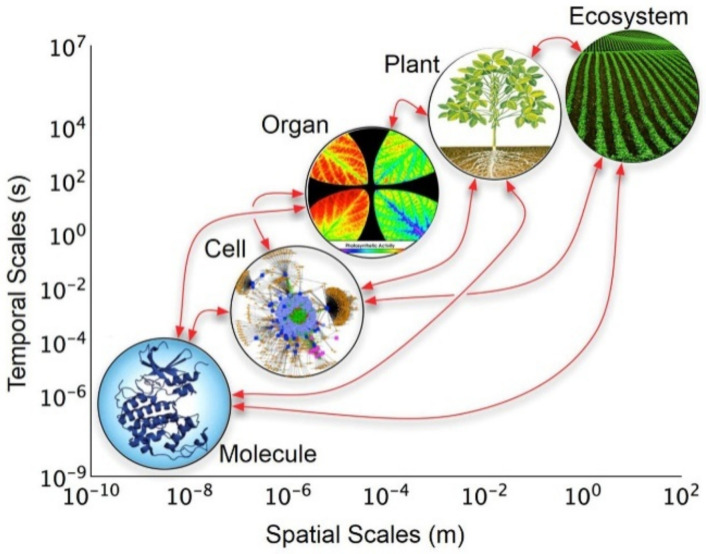
The holistic approach of hierarchical levels of organization of biological models across temporal and spatial scales. Reprinted with permission from reference [47]. Copyright 2017 Frontiers Media.

The rational design of methodological approaches in the quest forsatisfactory explanations of conceptual and empirical phenomena is the essence of holism [41,42]. In this context, it is highly relevant to understand the close relationship that exists in each of the parts that make up a given system and their interaction to form a whole [43]. Undoubtedly, this means that a whole is made up of parts, but the action that it exercises or performs cannot be unequivocally determined as the sum of its parts. This concept leads to thinking that the relationships and interactions between the parts contribute strongly to determining the qualities and attributes of the whole as a unit (i.e., the components are not independent of each other). In the historical context, the concept of holism in science was introduced in the second half of the 1920s, explaining the behavior of complex biological systems [44]. Considering this, in nature, it has been observed that the interaction among the component parts of a living organism (e.g., cells) acting as a whole allows for the preservation of their existence (Figure 2) [45,46,47].

**Figure 3:**
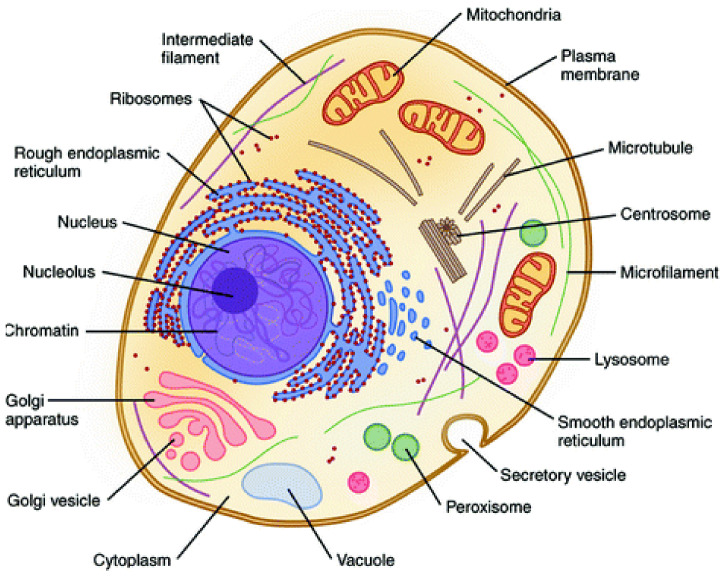
Representative structure of the cell showing their constituent organelles. Reprinted with permission from reference [50]. Copyright 2020 Springer.

Therefore, the hierarchical interactions present in biological systems allow them to act as a whole, being more than the simple sum of their parts (i.e., hierarchical holism) [48,49]. Illustratively, inside the cell membrane, this behavior can also be observed because the coordinated interaction of their organelles (e.g., ribosomes, nucleus, mitochondrial) is fundamental for satisfying all needed biological functions (Figure 3) [50].

**Figure 4:**
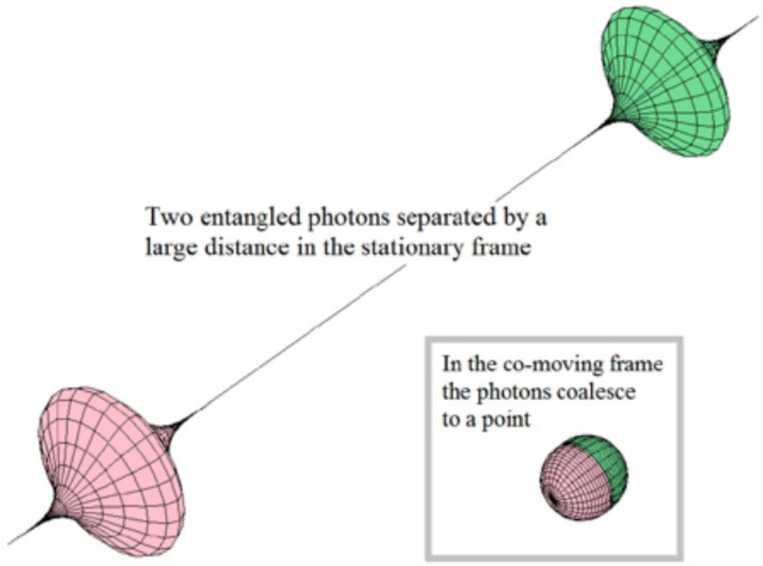
Illustration of two entangled photons. The state of one of them is instantaneously transferred to the other one. Reprinted with permission from [51]. Copyright 2015 Serials Publications.

A contrasting example is found in physics. The so-called entangled states belonging to quantum theory are often considered as an exception to the holistic principle. In this case, it is established that every system or physical entity can be described from its kinematic properties (e.g., velocity, position coordinates) in aform independent of other systems, i.e., a reductionist perspective. However, it is known that these properties can become dynamic (acting as more complex systems) when the influence of interactions with other systems is taken into account (Figure 4) [51]. Consequently, in the case of composting systems, the study of the state of an aggregate system is approached from the analysis of the states of the subsystems and their interactions.

**Figure 5:**
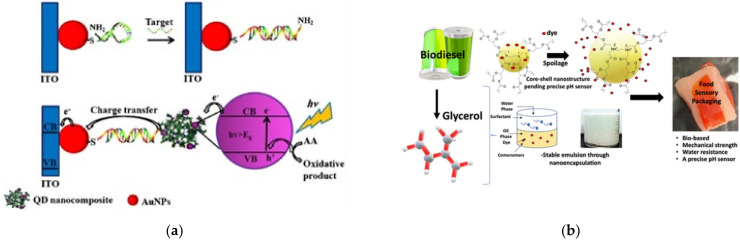

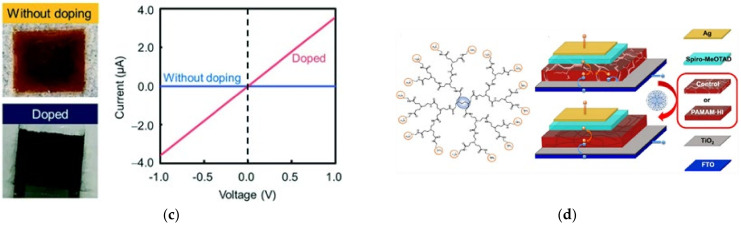
Dendrimer-based systems act as materials for (**a**) photocurrent generation, (**b**) sensor food spoilage detection, (**c**) semiconducting devices, and (**d**) enhancing the power conversion efficiency of solar cells. Reprinted with permission from [58] Copyright 2019 Springer; [59] Copyright 2021 American Chemical Society; [60] Copyright 2020 Royal Society of Chemistry; [61] Copyright 2021 Springer.

On the other hand, the importance of integrated systems is closely related to the rapidly emerging field of nanoscience and nanotechnology [52,53]. Nanotechnology addresses the rational design and potential technological applications of functional structures that generally exhibit a high degree of organization at the molecular and supramolecular scale [54,55]. Importantly, an integrated molecular system can be considered as hierarchical assembled molecules or macromolecules arranged to carry out specific processes [56,57]. This is the case already described for biological systems (e.g., mitochondria, the photosynthetic system). Other comparatively less structurally complex but sophisticated artificial systems are abundantly reported in the literature, such as photoelectrochemical systems [58], sensors [59], semiconductors [60], and devices for solar cells [61], among others (Figure 5).

These are all representative examples of integrated molecular systems that constitute a considerable amount of knowledge and experience accumulated over several years towards the development of nanotechnology. For example, many efforts have been devoted to designing more thermodynamically efficient integrated photochemical systems, allowing the conversion of light energy into clean, abundant, and highly usable energy for technological purposes [62,63]. As mentioned above, this approach is based on mimicking, to some extent, the function and organization of biological systems on a scale of similar dimensions (e.g., micro, nanoscale). In general, these types of artificial systems have sophisticated structures and architectures, with a high degree of organization (i.e., assembled materials) [64]. From the perspective of technological advances, the fabrication of self-assembled structures (through intermolecular interactions) is an important drive towards designing photoresponsive systems [65]. This is a relevant point, since the challenge of mimicking more complex processes or structures that turn out to be more efficient is an interdisciplinary challenge fully in force to date [66,67]. Therefore, the scientific and technological endeavors should feel strongly encouraged to continue providing valuable efforts to better understand the role of functional holistic-integrated molecular systems [68,69].

## 3. Photo-Induced Energy Transfer Processes

Photo-induced energy transfer (PhET) involves light-induced processes present in molecular systems that contain photoactive chemical entities (e.g., chromophore groups) [70]. Globally, this type of process requires the study and an adequate understanding of the behavior of the excited states of organic molecules. Importantly, the thorough analysis of these processes can evidence valuable information on the light-induced energy-transfer mechanisms [71].

PhET processes are a highly relevant phenomenon both in nature and in artificial systems. A representative case is photosynthesis, wherein numerous energy transfer phenomena occur in the reaction center [72,73]. This triggers different mechanisms of ion separation and charges transport for the effective synthesis of energetic molecules. Thereby, photosynthesis can be visualized as large and complex machinery for converting light radiation into chemical energy [74]. In the case of artificial systems, photofunctional materials should exhibit attributes to harvest and convert solar energy into usable energy. Considering this, several examples can be cited as suitable molecular systems to favor photon-induced energy transfer, such as micelles [75], vesicles [76], colloids [77], monolayers [78], and dendrimers [79].

The above-mentioned systems require incorporating photoexcitable molecules or entities by hosting into their nanoreservoirs, intermolecular interactions, or forming covalent bonds. Ideally, photoexcitable molecules must contain active energy sites, donors, and acceptors. Additionally, the chemical environment would favor and stabilize the photoexcited species involved in the energy transfer process. Note that these phenomena occur at nanoscale dimensions; hence, the dimensions of the inner reservoirs of the host systems should be on that magnitude [80,81]. Considering these aspects, dendrimer-based systems can be conceived as a proper medium to ensure that the non-radiative process is raised for the lifetime of a fluorophore [82]. It is important to mention that the light-harvesting process performed by photoactive systems bearing electron-donor active sites involves the unidirectional transfer of the absorbed radiation energy [83]. This is an essential condition observed in nature, where photoactive centers composed of numerous chromophores exhibit a specific position or determine the spatial orientation for each other [84]. Therefore, artificial chemical systems would contain several symmetrically distributed light-harvesting donor–acceptor sites (into micellar, vesicular, or dendrimer structures) to appease a directional energy transfer over nanoscale dimensions. In recent years, the fluorescence (or Förster) resonance energy-transfer (FRET) mechanism has been studied in-depth to concentrate absorbed energy at a spatially confined site in nanometric regions [85,86,87]. FRET systems involve the presence of a significant amount of chromophores able to mainly absorb light radiation, e.g., in the visible region of the spectrum (this is addressed in more detail below). Importantly, these artificial systems would be suitable for generating multi-step mechanism directional energy transfer of the absorbed radiation energy [88]. These chemical environment conditions would allow for reaching photoactive centers separated by distances in the order of nanometers. Additionally, it is expected that the energy of photoexcitation of the chromophore centers corresponds typically to S_0_→S_1_ transitions (i.e., from HOMO to LUMO) [89,90].

### 3.1. FRET Phenomenon

**Figure 6:**
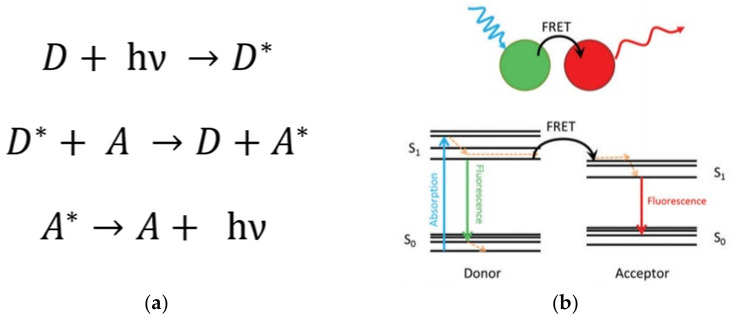
(**a**) Description of the FRET mechanism. D (A) and D* (A*) are the ground state and excited state of the donor unit (acceptor unit), respectively; *h* is the Planck’s constant; *ν* is the frequency of the radiation. (**b**) Representation of the FRET phenomenon by Jablonsky diagrams. The excited state of the donor unit (spatially close to the acceptor unit) reaches relaxation by the FRET mechanism exciting the acceptor (non-radiative energy transfer). The relaxation stage of the acceptor unit is evidenced by the fluorescence process emitting light. Note that the emitted light is a lower wavelength than the emission of the donor in a regular fluorescence process. Reprinted with permission from [92]. Copyright 2016 SAGE Publications.

This phenomenon, studied by Theodor Förster, consists of the transfer of light energy from an excited donor (D) unit to an acceptor (A) group through a non-radiative process (Figure 6) [91,92]. Importantly, this process is profited to estimate the separation distance at the molecular level between chromophores, such as that between a donor–acceptor pair. This is valid, mainly for separation distances smaller than 10 nm [93,94]. Thus, this phenomenon is highly sensitive to the spatial dimensions and specific orientation and the chemical nature of the environment surrounding the chromophore units. Thereby, FRET can be used as a reliable technique to characterize the dynamics, e.g.,the conformational changes of macromolecules, helping to establish the nature of intermolecular interactions in natural (e.g.,biological molecules) and artificial (e.g., micelles, dendrimers) nanosized systems having chromophores groups [78,95,96,97].

A more detailed theoretical description of this mechanism consists of a donor entity reaching an excited state due to interaction with a photon. Subsequently, according to the Kasha rule, the donor chromophore undergoes a relaxation process to the lowest excited singlet state (S_1_). Importantly, the acceptor chromophore group, which must be at an average distance no larger than 10 nm, absorbs the released energy (undergoes a simultaneous excitation process) when the electron returns to the ground state of the donor chromophore (S_0_). Importantly, this non-radiative process that occurs through a dipole–dipole coupling is called “resonance energy transfer” [98,99]. Lastly, the exciting electron from the acceptor chromophore group returns to the ground state S_0_, releasing a photon.

**Figure 7:**
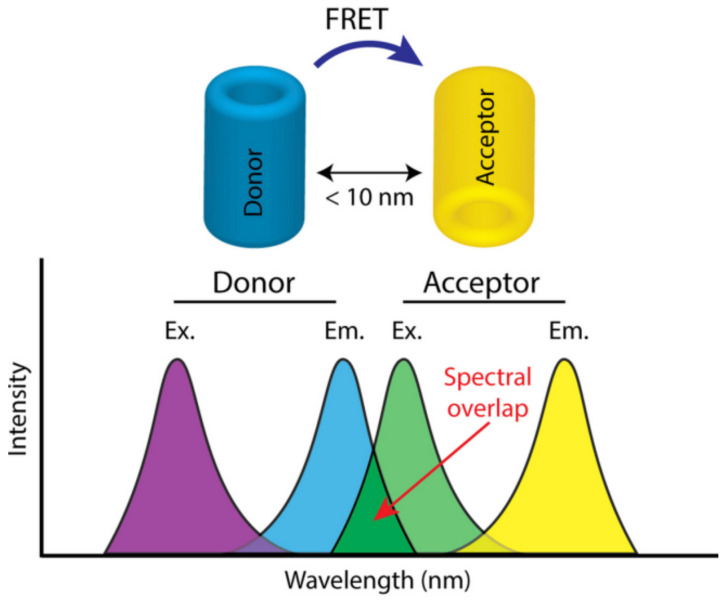
The density of states obtained from the spectral overlap between donor-emission (Em.) and acceptor-excitation (Ex.) bands. The efficiency of the FRET phenomenon is maximizedwhen the donor and acceptor molecules are at an average distance of 10 nm from each other, and their respective dipole moments are oriented in parallel. Reprinted with permission from [101]. Copyright 2017 Elsevier.

Note that non-radiative energy transfer mechanisms are strictly related to the lifetime of the excited state of the donor species (the duration of this state). In this case, the formation of a transfer complex occurs through the interaction of the excited donor and the acceptor in the ground state, as long as there is a proper distance between the species. According to Fermi’s golden rule for independent systems models, energy transfer rates (*k_ET_*) between a donor and an acceptor unit can be expressed as [100]:

$$k_{ET}=\frac{2\pi }{\hbar }\left|\langle DA^{*}\rangle \right|H{\left|D^{*}A\right|}^{2}\;\rho \left(E_{DA^{*}}\right)$$

where $|D^{*}A\rangle$ and $\langle DA^{*}|$ represent the initial state of the complex (before the energy transfer, $D^{*}A$) and the final state of the complex (after the energy transfer, $DA^{*}$), respectively. $\rho \left(E_{DA^{*}}\right)$ is the density of states at the $E_{DA^{*}}$, the energy of the final state. This parameter can, generally, be obtained from the spectral overlap between donor emission and acceptor excitation bands (Figure 7) [101]. Additionally, *H* is the Coulomb interaction hamiltonian(a harmonic perturbation) between the electronic clouds and an acceptor [102].

As delineated, the energy-transfer process initially involves a Coulomb interaction between the electronically excited state of the donor and the electronic ground state of the acceptor (i.e., overlap or wavefunctions). Subsequently, the interaction occurs between the acceptor’s electronically excited state with the donor’s ground state. In this case, the Coulomb interaction is feasible since, in energetic terms, the emission process of the donor chromophore is comparable to that of absorption of the acceptor unit [103,104]. Thereby, the eventual transfer of energy for this absorption and emission process occurs simultaneously. Note that the Coulomb interactions between the electronic states of both the donor and the acceptor occur during the lifetime of the donor fluorescence process and depend on the distance (r) of the donor and acceptor units by a factor r^−3^ [105,106,107]. Additionally, the probability that the energy-transfer process occurs is proportional to the square of the distance, i.e., r^−6^ [108]. According to Förster’s expression, the energy transfer rate (*k_DA_*) is given by [109,110]:

$$k_{DA}=\frac{\kappa^{2}k_{r}^{D}}{R^{6}n^{4}}\;\cdot 8.8\;\times \;10^{17}\;\cdot \;\overset{\infty }{\underset{0}{\int }}\frac{\epsilon_{A}(\nu )\;\cdot \;f_{D}(\nu )}{\nu^{4}}\;d\nu$$

$k_{DA}$ (ps^−1^), $k_{r}^{D}$ (ps^−1^), R (nm), $\epsilon_{A}(\nu )$ (M^−1^ · cm^−1^), *ν* (cm^−1^)

where $k_{DA}$ is the transfer rate between donor and acceptor entities, *κ* is the orientation factor, *n* is the refractive index, *R* is the distance between the centers of the interacting donor and acceptor units, and $k_{r}^{D}$ is the radiative rate of the donor entity. Considering the terms within the integral, ν is the frequency, $\epsilon_{A}(\nu )$ is the absorption molar coefficient of the acceptor unit, and $f_{D}(\nu )$ is the normalizeFd emission spectrum of the donor entity. It is important to mention that the integral term reflects the overlap between the normalized fluorescence spectrum (per unit area) of the donor and the extinction coefficient at the maximum absorption of the acceptor, both as a function of frequency.

Furthermore, κ is given by [111,112]:

$$\kappa =\;\left({\hat{\mu }}_{D}\;\cdot \;{\hat{\mu }}_{A}\right)\;-\;3\left({\hat{\mu }}_{D}\;\cdot \;{\hat{r}}_{DA}\right)\left({\hat{\mu }}_{A}\;\cdot \;{\hat{r}}_{DA}\right)$$

where, ${\hat{\mu }}_{D}$ and ${\hat{\mu }}_{A}$ are the normalized vectors of the transition dipole moments of donor and acceptor units, respectively, and ${\hat{r}}_{DA}$ is also a normalized vector related to the distance between the geometric centers of the donor and the acceptor.

Importantly, if the probability of occurrence of this transfer process is 50%, it is interpreted as a critical distance (known as the Försterradius, *R*_0_) [113,114]. This critical distance is considerably longer than the average bond distance; hence, this process is considered a long-distance energy transfer. More specifically, this radius is defined as the distance where the transfer rate, $k_{DA}$, is equal to the lifetime (*τ_D_*) of the donor fluorescence process determined in the absence of an acceptor entity. According to this, the mathematical expression to describe the above-mentioned phenomenon is as follows [115,116]:

$$k_{DA}\;=\;\frac{1}{\tau_{D}}\left(\frac{R_{0}{}^{6}}{r^{6}}\right)$$

Here, by measuring the FRET efficiency, a sufficiently accurate estimate of the distance between the donor and acceptor chromophores can be obtained. Even so, there are some limitations in the application of this model. For example, the conformational changes, namely, the folding or unfolding of macromolecules, should be moderate in length scale (≤10 nm); otherwise, it is not possible to attribute the positional changes of the chromophores involved [117,118].

### 3.2. Dendrimer-Based Molecular Systems for FRET Phenomenon

Currently, various research areas, focused on physics, chemistry, engineering, and biotechnology, among others, have the primary objective of taking advantage of solar radiation as a source of clean and abundant energy [119,120,121]. Solar-energy conversion begins with the collection of light. This requires the continuous development and synthesis of new advanced synthetic materials to harvest photon energy, eventually converting it and efficiently transferring it to other active sites. The efficiency of this process depends on the spatial distance (e.g., between donor–acceptor sites) of the transfer of light energy to the site where it can be transduced into other forms of energy [122,123,124].

Given the above, it is intended that the characteristics and attributes exhibited by different artificial chemical systems allow for the mimicking of the complex architectures and structures present in nature and the function they fulfill in light-induced processes [125,126]. In this context, dendrimers emerge as excellent candidates since they can play a highly relevant role as unimolecular reactors at the nanoscale, acting as versatile and sophisticated entities [127,128]. These systems can be suitable for performing synchronously as a whole unit, integrating donor and acceptor chromophoric units in their structure [129]. In addition, their well-defined branched architectures would allow for various chromophore arrays with relative positions and orientations to create one-step or multi-step gradients (cascades) of energy and spatial focalization of the excitation energies [130]. These are relevant aspects since they condition the system’s behavior concerning the harvesting of light and the efficiency of the energy-transfer processes. The above-mentioned factors constitute a search that is mainly inspired by mimicking and a better understanding of the design and functions of complex systems present in nature, motivating intense research in recent decades [131,132].

**Figure 8:**
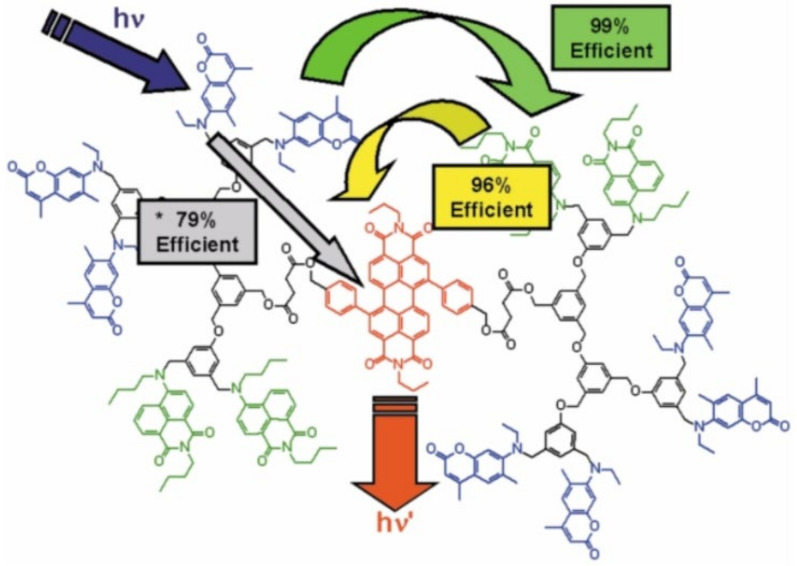
Representation of FRET efficiencies between chromophores using, as an initial donor, coumarin (**blue**), an intermediate fluorol (**green**), and a final acceptor perylene (**red**). Reprinted with permission from [135]. Copyright 2002 Royal Society of Chemistry.

Subsequently, the use of dendrimers for those purposes has made it possible to suppress, or at least control, undesirable phenomena, such as chromophore self-quenching and excimer species formation, among others [133,134]. For this, conformationally flexible dendrimers of poly (aryl ether), generations 2 and 3, turned out to be highly useful in the transfer of resonance energy from the lifetime of the fluorescence state of the donor species avoiding the formation of excimers [135]. In this way, it was possible to promote the occurrence of the FRET phenomenon to concentrate the absorbed energy in a single photoactive center (Figure 8) [136]. In this seminal work, the unidirectional cascade FRET phenomenon in these conformationally flexible systems was studied [137]. It was revealed that the energy transfer process in this type of system is favored through the vectorial cascade route [138]. The above can be understood as a sequence of chained processes, in which the energy transfer occurs from an initial donor chromophore (coumarin), passing through an intermediate chromophore (fluorol) until it reaches a final acceptor chromophore (perylene). To demonstrate this behavior, the authors conducted rigorous photophysical analyzes of the steady states (absorption and emission behavior) of dendrimer-based systems, which allowed the FRET efficiencies to be estimated. Interestingly, the emission spectra’s information helped to establish the absence of other competing phenomena, such as aggregation, excimers, or the self-quenching of donor chromophores in the emission process [139,140].

**Figure 9:**
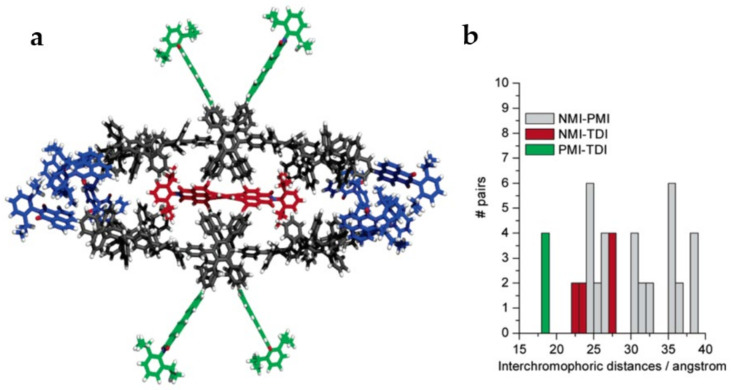
(**a**) Illustration of the energetically minimized structure of the triad constituting naphthalenemonoimide(blue), perylenemonoimide(green), and terrylenediimide(red) moieties. (**b**) Center-to-center interchromophoric distances calculated from the energetically minimized structure. Reprinted with permission from [143]. Copyright 2005 American Chemical Society.

Similarly, studies on cascade light-harvesting phenomena using conformationally rigid dendrimers have also been carried out. An environment with higher rigidity could likely contribute to reducing the energy losses resulting from the short-distance interactions (<10 nm, giving rise to the appearance of competing phenomena) among chromophores [141]. For example, highly rigid dendrimers composed of three condensed aromatic systems acting as a multi-chromophoric system were synthesized [142]. Notably, these chromophores exhibit different absorbance and emission behaviors, covering the entire visible spectrum.For this, a rational synthetic strategy using terrylenediimide(TDI) as a core, functionalized with four dendrons of perylenemonoimides(PMI) and eight naphthalenemonoimides(NMI) units located at the periphery, was employed (Figure 9) [143].The relative orientation, along with the intrinsic photophysical properties of the triad, allowed it to perform as an efficient visible-light-harvesting multi-chromophoric system. Importantly, two energy-transfer mechanisms were detected, depending on the species present: (i) unidirectionally or (ii)cascading towards the acceptor core. It is important to mention that the eventual presence and contribution to the process of some oxidized species of chromophores could influence the mechanism that occurs [144]. In the final stage, this energy was released into the medium as red fluorescence by the acceptor unit [145].

The chemical groups present in the dendrons and the core and the generation of the dendrimer play a key role in favor of the feasibility of the FRET phenomenon [146]. For example, time-resolved and steady-state measurements showed that π-conjugated dendrimers (generations 0 and 2) made up of styrylbenzene groups exhibit a fast (~7 ps) and highly efficient energy transfer (close to 100%) from the dendrons to the acceptor site, an amino group located in the nucleus [147]. Accordingly, the course of this process can adequately be described considering the Förster mechanism. Strongly π-conjugated systems displaying well-defined branched geometries and having an acceptor as the core of the dendrimer significantly influenced the intermolecular interactions, which intervene in the efficient transfer of non-radiative energy [148].

**Figure 10:**
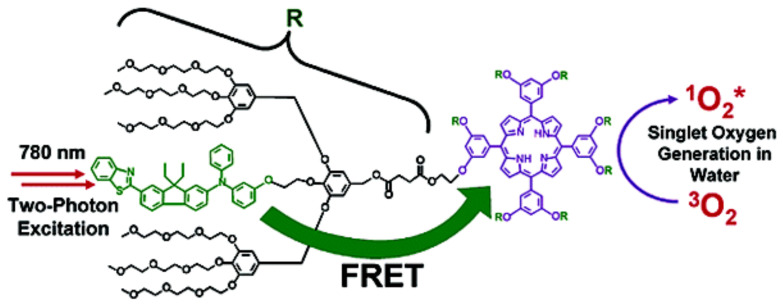
Mechanism of singlet oxygen photosensitization via indirect excitation of the photosensitizer (porphyrin) by two-photon-excited FRET from distanced chromophores present into a dendrimer. Reprinted with permission from [149]. Copyright 2005 American Chemical Society.

Inspired by the singlet oxygen species produced through the light-harvesting process by chlorophyll molecules in photosynthesis, Oar et. al [149] (Figure 10) designed water-soluble dendrimeric photoactive systems to increase the generation and propagation of reactive oxygen species (ROS). Singlet oxygen is a chemical species of high interest since its efficient generation could be useful in various technological developments, such as the photocatalytic degradation of polluting species present in various sources. In photodynamic therapy and tissue imaging, the generation of ROS would help to reduce the limitations depth that exists in different types of living tissue and to obtain an enhanced spatial resolution [150]. The strategy that was carried out contemplated using indirect excitation of spatially separated chromophores present in the dendrimer structure by two-photon-excited fluorescence resonance energy-transfer (FRET). In this technique, the donor entity is excited with two photons with wavelengths belonging to the near-infrared (NIR) spectrum. The two-photon absorbing donor chromophores were covalently bonded to the periphery of the dendrimer structure. Furthermore, porphyrin is located at the core of the dendrimer, which acts as an acceptor chromophore and a singlet oxygen-generating species. The authors demonstrated an exceptionally high FRET efficiency (>99%) between the peripheral donor chromophores and the central acceptor, caused by the transfer of fluorescence resonance energy excited by two photons in aqueous medium. 

The incorporation of metal centers as acceptor sites is an innovative strategy to combine the inherent virtues of organic and inorganic compounds. In this sense, it is well documented that various ruthenium complexes (especially coming from bipyridine family, bidentate chelating ligands) are highly usable systems for processes requiring significant photoactivity [151]. Simultaneously, four covalently bonded multi-chromophoric dendrons to the metal center would give the dendrimer rigidity (almost unaltered distance) and the unidirectionality of non-radiative energy transfer at scales of molecular dimensions necessary to promote the FRET phenomenon [152]. The literature analysis reveals that the integration of this type of system in devices in the solid phase can be attained. Thus, a possible application in the field of photovoltaic energy is highly exploitable, as it would increase sensitization for the light-harvesting in the red and near-infrared region of the solar spectrum [153,154].

**Figure 11:**
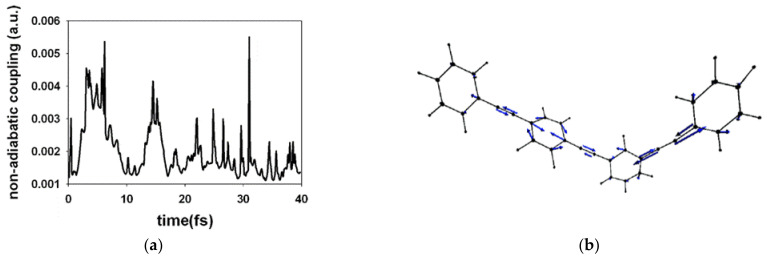
(**a**) Averaged values of the overall trajectories of non-adiabatic coupling vector of S_2_→S_1_ hop and (**b**) illustration of the effective hop process in the dendron at ≈ 5 fs. Reprinted with permission from [155]. Copyright 2009 American Chemical Society.

Alternatively, theoretical studies based on molecular simulation dynamics helped to deepen in detail the ultrafast process of vibrational energy transfer between two- and three-ring linear phenylene ethynylene dendrons (PPE) [155] (Figure 11). Specifically, the analysis shed light on the fact that the transfer of the electronic state population from the S_2_ state to the S_1_ state is related to the ultra-fast transfer of vibrational energy from the two-ring system to the three-ring system [156]. After the photoexcitation process, a rapid decrease in the energy gap between the S_1_–S_2_ levels was observed, which resulted in the transfer of the electronic population S_2_ → S_1_. In addition, it was estimated that the depopulation time of the S_2_ level, in about 60%, occurs at approximately 40 fs. Importantly, the trajectory analysis revealed that the stretching motions in the direction of the bonds of the ethynylene units strongly favor the unidirectional energy transfer process. In this sense, this type of system establishes, as a requirement, the change in the stretch lengths of the ethynylene bonds. This produces an efficient and ultra-fast unidirectional transfer of vibrational energy (i.e., non-radiative) two-ring entities→three-ring entities [157].

Importantly, experimental studies, combined with theoretical analyses, allowed researchers to delve into ultra-fast and unidirectional electronic energy transfer processes in complex systems designed to spatially orient the initial excitation in an energy sink environment [158]. The analysis of the population of the excited state and the distribution of the excitation energy of the atoms belonging to π-conjugated dendrons composed of phenylene-ethynylene (PE) units allowed us to infer that the mechanism of electronic energy transfer involves the ultra-fast collapse of the photoexcitation wave function due to non-adiabatic electronic transitions [159]. In this dendrimer system, there is a significant density of relatively delocalized coupled excited states produced by the initial excitation energy (thermal disorders could also contribute). Interestingly, the significant coupling of high-frequency vibrational modes promotes non-adiabatic electronic transitions [160]. Simultaneously, the excess electronic energy is converted into nuclear motions spread throughout the molecule (loss of anisotropy). In this way, it is proposed that dynamic phenomena of a non-adiabatic nature were essential for π-conjugated systems [161]. For this type of entity, a strong contribution to the ultrafast dynamics of excitation localization by appreciable densities of excited states, strong exciton–phonon coupling, and relevant thermal fluctuations would be highly favored [162].

The use of dendrimeric systems to study the transfection process in cells with a view to therapeutic applications has been recently studied. Importantly, the synthesis of dendrimers composed of peptide blocks as transfection agents combined with genetic material (RNA) is proposed as a promising strategy. Incorporating fluorescent molecules (e.g., coumarins) in the nucleus of this type of dendrimers makes it possible to monitor the dendrimer–nucleic acid association phenomena through the FRET mechanism [163]. Specifically, it seeks to deepen the understanding of the transfection process by directly measuring the interaction or complexation between dendrimers and nucleic acids, enabling their location within cells. These studies open an innovative perspective to enable the monitoring of the transfection process within cells using, for example, fluorescence microscopy [164]. Furthermore, it is highlighted that the transfection of cells using peptide dendrimers resulted in a wide variety of hydrophobic nuclei. In this type of analysis, peptide dendrimeric systems have several advantages beyond monitoring the process, such as good viability of the synthesis route, low toxicity, and high transfection efficiency in different cell lines [165].

**Figure 12:**
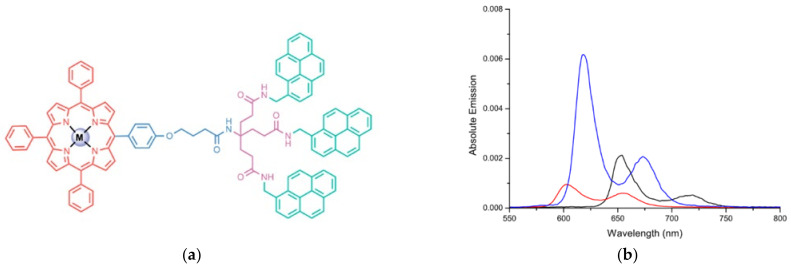
(**a**) Chemical structure of first-generation pyrene dendronized porphyrins having a metal center and (**b**) absolute emission spectra recorded in THF for the metal-free (black line) and metalated porphyrins (M = Zn and Mg are represented by red and blue lines, respectively). Reprinted with permission from [166]. Copyright 2021 Elsevier.

Conveniently, the metallation of dendrimer centers of different generations having a molecule suitable (e.g.,porphyrins)forhosting metal ions and pyrene molecules at the periphery has been developed to study their absorption and emission behaviors. The porphyrinicdendrimeric center’s complexation of Zn (II) and Mg (II) ions resulted in a red and blue shift of the absorption and emission bands, respectively [166] (Figure 12). Notably, this dendrimers type showed high non-radiative energy-transfer efficiency, reaching values higher than 99%. According to these results, it can be inferred that the combination of the redox properties of metal ions, along with the photoactivity of organic molecules, significantly enhances the FRET mechanism [167,168]. Interestingly, the mimicking of the role, structure, and properties of molecular systems present in nature (e.g., chlorophyll) can be seen, to some extent, reflected [169].

Very recently, a novel strategy has been used to prepare complex coacervated core micelles by compatibilizing four types of dendrimers containing different cores [170]. Studies show that these mesoscale structures have a hydrodynamic radius and a core size on the nanometer scale. Interestingly, these complex structures can host a significant number of high-generation dendrimers in the nucleus region. Dendrimers subjected to confinement in the nucleus can be functionalized with fluorophores such as fluorescein and rhodamine. Interestingly, the analysis of the FRET phenomenon granted the possibility of monitoring the synchronous encapsulation process of the dendrimers in the micellar nucleus.

Additionally, the FRET mechanism revealed two important aspects: (i) it is possible to modulate the proximity between dendrimers, mainly by adjusting the feeding ratio of the dendrimeric systems and their co-assembly before confinement in the nucleus; and (ii) the existence of a minimum loading of dendrimers in the core to achieve optimal FRET efficiency [171,172]. The design of this type of advanced materials makes it possible to manufacture multicompartmental nanoreactors (synthesis of stable nanocarriers) and to use them as fluorescent tracers in biomedical applications for diagnostic therapies [173,174].In recent decades, the scientific and technological interest in mimicking or replicating, to some extent, the molecular-level arrangements that exist in nature for the capture of light energy (e.g., photosynthesis, phototropism) has attracted considerable attention. In this sense, dendrimers have emerged as an innovative alternative due to their interesting structural properties and their high versatility and functionality. The unimicellar architecture of this type of system shows high chemical adaptability, allowing localized covalent functionalization with chromophores for use as light-energy harvesters. The versatility and regular distribution of functional groups on the periphery allow for the incorporation of multiple light-energy harvesting centers that can transfer it, for example, to the dendrimer core.

## 4. Future Perspectives

Essentially, this compendium of studies embodies (Table 1) a concise overview of the past two decades of a relevant technological application of chromophoric dendrimer systems. In particular, it is seen that the unique architecture and the abundant chemical functionality, mainly in the core and at the periphery of the dendrimers, play a key role in the properties of light-energy harvesting and non-radiative energy transfer. In this way, this type of molecular system integrates different chemical entities and their respective attributes, allowing them to function as a whole unit. Considering these antecedents, the phenomena of light absorption and the subsequent transfer of non-radiative energy in a unidirectional and spatially focused way could be highly exploited for varied uses, such as optoelectronic devices, fluorescent sensors, imaging markers, molecular detection, energy storage, and so on. Importantly, various reports show a high-efficiency transfer of energy in dendrimeric systems, mimicking processes that occur in nature. From the holistic-integrated perspective, dendrimers can act as a whole-system approach with photo-optical properties, tuned by synthesis routes, chemical modifications of the nucleus or the periphery, types of dendrons, and generation, among other possibilities.

## 5. Concluding Remarks

### 5.1. General Outlook

In recent decades, the scientific and technological interest in mimicking or replicating, to some extent, the molecular-level arrangements that exist in nature for the capture of light energy (e.g., photosynthesis, phototropism) has attracted considerable attention. In this sense, dendrimers have emerged as an innovative alternative due to their interesting structural properties and their high versatility and functionality. The unimicellar architecture of this type of system shows high chemical adaptability, allowing localized covalent functionalization with chromophores for use as light-energy harvesters. The versatility and regular distribution of functional groups on the periphery allow for the incorporation of multiple light-energy harvesting centers that can transfer it, for example, to the dendrimer core.

### 5.2. DendrimericSystems Attributes

The distance and relative orientation between donor and acceptor chromophores play a key role in the process of non-radiative energy transfer. In this aspect, the modulation of the flexibility or rigidity of dendrimer systems can significantly contribute to the unidirectional transfer of the absorbed radiation energy. Remarkably, dendrimers exhibitingππ-conjugated bonds bearing metal complexes on the periphery or metalated centers influence the efficiency of the non-radiative energy transfer. This was corroborated by theoretical studies, which demonstrated that the above-mentioned structural characteristics are involved in the fast energy transfer of the photoexcited species due to non-adiabatic (e.g., non-radiative decay after photoexcitation) electronic transitions.

### 5.3. Technological Applications

Finally, an in-depth understanding of the FRET mechanism in diverse types of dendrimeric systems allows for diverse and prolific technological applications, such as imaging, biomedical therapy, and the conversion and storage of light energy, among others.

## Figures and Tables

**Table 1:** Summary of the main topics addressed by reviewed studies.

Author	Main Topic	Reference
Serin et al.	Unidirectional cascade FRET phenomenon in conformationally flexible systems.	[135]
Wu et al.	Highly rigid dendrimers composed of three condensed aromatic systems, acting as a multichromophoric system.	[143]
Varnavski et al.	Time-resolved and steady-state measurements showed that π-conjugated dendrimers exhibit a fast and highly efficient energy transfer.	[147]
Oar et. al	The strategy carried out contemplated using indirect excitation of spatially separated chromophores present in the dendrimer structure by two-photon-excited fluorescence resonance energy transfer (FRET).	[149]
Carrone et al.	Incorporation of metal centers (rutheniun) as acceptor sites. Specifically, ruthenium complexes coming from bipyridine family exhibited considerable photoactivity.	[151]
Fernandez-Alberti et al.	Theoretical analysis shed light on the transfer of the electronic state population from the S_2_ state to the S_1_ state is related to the ultra-fast transfer of vibrational energy from the two-ring system to the three-ring system.	[155]
Geng et al.	Experimental studies combined with theoretical analyses allowed them to delve into ultra-fast and unidirectional electronic energy-transfer processes in p-conjugated systems designed to spatially orient the initial excitation in an energy sink environment.	[158]
Heitz et al.	The incorporation of fluorescent entities in the nucleus of dendrimers (to study the transfection process in cells) makes it possible to monitor the dendrimer–nucleic acid association phenomena through the FRET mechanism.	[163]
Bañales-Leal et al.	Porphyrinic dendrimers containing Zn (II) and Mg (II) ions showed high non-radiative energy-transfer efficiency, reaching values higher than 99%.	[166]
Kaup et al.	Preparation of complex coacervated core micelles by compatibilizing four types of dendrimers containing different cores and fluorophores. The FRET phenomenon granted the possibility of monitoring the synchronous encapsulation process of the dendrimers in the micellar nucleus.	[170]

## Data Availability

Not applicable.
